# Breast conserving surgery versus mastectomy: cancer practice by general surgeons in Iran

**DOI:** 10.1186/1471-2407-5-35

**Published:** 2005-04-05

**Authors:** Massoome Najafi, Mandana Ebrahimi, Ahmad Kaviani, Esmat Hashemi, Ali Montazeri

**Affiliations:** 1 Iranian Center for Breast Cancer (ICBC), Tehran, Iran; 2 Department of Surgery, Faculty of Medicine, Tehran University of Medical Sciences, Tehran, Iran

## Abstract

**Background:**

There appear to be geographical differences in decisions to perform mastectomy or breast conserving surgery for early-stage breast cancer. This study was carried out to evaluate general surgeons' preferences in breast cancer surgery and to assess the factors predicting cancer practice in Iran.

**Methods:**

A structured questionnaire was mailed to 235 general surgeons chosen from the address list of the Iranian Medical Council. The questionnaire elicited information about the general surgeons' characteristics and about their work experience, posts they have held, number of breast cancer operations performed per year, preferences for mastectomy or breast conserving surgery, and the reasons for these preferences.

**Results:**

In all, 83 surgeons returned the completed questionnaire. The results indicated that only 19% of the surgeons routinely performed breast conserving surgery (BCS) and this was significantly associated with their breast cancer case load (P < 0.01). There were no associations between BCS practice and the other variables studied. The most frequent reasons for not performing BCS were uncertainty about conservative therapy results (46%), uncertainty about the quality of available radiotherapy services (32%), and the probability of patients' non-compliance in radiotherapy (32%).

**Conclusion:**

The findings indicate that Iranian surgeons do not routinely perform BCS as the first and the best treatment modality. Further research is recommended to evaluate patients' outcomes after BCS treatment in Iran, with regard to available radiotherapy facilities and cultural factors (patients' compliance).

## Background

Many randomized clinical trials have demonstrated that patient survival rates are similar after treatment by mastectomy or by conservative surgery and radiotherapy [1-4]. However, the National Institute of Health Consensus Conference concluded that for most women with early-stage breast cancers (stages I and II), breast conservation surgery (BCS) is an appropriate method of treatment [5]. Despite these findings, mastectomy remains the most prevalent surgical treatment for early-stage breast cancer in many parts of the world. In Iran, BCS is an uncommon modality for this condition. For example, in one study in Isfahan University of Medical Sciences, 386 breast cancer patients were reviewed and mastectomy was found to be the most prevalent surgical treatment [6]. Similarly, in another study in Tehran University of Medical Sciences, 348 breast cancer patients of whom 50% had stage I or stage II breast cancer were evaluated, and it was found that only 22 patients underwent BCS [7].

It is argued that one factor influencing choice of treatment in breast cancer patients is the attitude of the operating surgeon. There is evidence that most women follow surgeons' recommendations, their primary source of information about treatment options. Studies have shown that surgeons recommended a specific treatment option in 69% of cases; in 89% of these cases they offered mastectomy and in 11% BCS. The rates of compliance with these recommendations were reported to be 93% and 89% respectively [8].

The present study was designed to evaluate the preferences of surgeons in Iran regarding breast cancer treatment and the factors influencing the type of treatment recommended.

## Methods

A descriptive study was conducted to investigate cancer practice among general surgeons in Iran. A specially designed questionnaire was mailed to a sample of 235 general surgeons, selected randomly from the Iranian Medical Council's address list for general surgeons and including a few surgical oncologists. All general surgeons (n = 1977, 1831 male and 146 female) belong to the Council. It is worth noting that in Iran, gynecologists and plastic surgeons do not perform breast cancer surgery. The sample size was determined by expectations of the rate of BCS treatment by the general surgeons: it was estimated that at best 15% and at worst 5% of the surgeons might perform BCS. Based on this assumption, a sample of 81 surgeons would give results with a 99% confidence level. To achieve this sample size it was decided to mail the questionnaire to 235 general surgeons, nearly three times more than needed. The questionnaire addressed the surgeons' characteristics including age, gender, work experience, posts held (teaching hospitals or private and public institutions), and number of breast cancer patients treated per year. In addition, respondents were asked whether they routinely performed BCS, and if the answer was "no" the reasons were solicited. Data were analyzed in a descriptive fashion, reporting numbers and percentages and using the chi-square test (Fisher's exact test) where necessary.

## Results

In all, 83 completed questionnaires were returned, giving a response rate of 33%. The characteristics of the study sample are shown in Table 1. The mean age of the respondents was 55.2 years (SD = 12.3; range 32 to 80). Most surgeons were male (n = 75, 90%), had more than 10 years work experience (76%), and were working at public or private centers (75%). For 80% of the surgeons surveyed, the average number of breast cancer patients treated per year was less than 20; 20% reported that their annual case-load was more than 20.

Only 19% of the surgeons said that they performed BCS routinely. Table 2 shows the reasons given for avoiding BCS. The most frequent reason was the surgeons' uncertainty about the results of breast conserving surgery (46.3%). The other most frequently-stated barriers to performing BCS were uncertainty about radiotherapy services and patients' non-compliance in radiotherapy (32.8%). Of the factors studied, only breast cancer case-load had a statistically significant association with performance of BCS (P = 0.007). The results are shown in Table 3.

## Discussion

Breast-conserving surgery is a treatment modality for early-stage breast cancer that causes less physical disfigurement and psychological trauma to the patient. Many prospective randomized trials have demonstrated that overall and disease-free survival rates for early-stage breast cancer are equivalent after mastectomy or BCS with postoperative radiotherapy [2-4], [8,9]. There appear to be geographical differences in the surgical treatment of early-stage breast cancer. Evidence suggests that physician recommendation exerts the most significant influence on surgical treatment selection for the majority of women [10].

In this study only 19% of surgeons used BCS in their practice while the remaining 81% performed mastectomy. This concurs with two other studies, performed in Tehran and Isfahan University of Medical Sciences, showing that mastectomy was the most prevalent surgical treatment for breast cancer patients [6,7]. However, it is arguable that BCS rates would rise in Iran if general surgeons, for example, could benefit from continuing medical education, attend scientific meetings, and have access to modern textbooks and current journals. Discussion of these topics lies beyond the scope of this study. Briefly, it should be noted that there is a National Society of Surgeons in Iran. In addition to other scientific meetings that address oncology issues, members of this society have their own annual and occasional scientific meetings, and usually receive printed educational materials including issues on current technology. At present all surgeons and radiation therapists in teaching hospitals and many working in the private sector are Board-certified. Some modern radiation therapy facilities in Iran are very similar to existing facilities in developed countries. Furthermore, we suspect that predicting patient non-compliance with radiation therapy might be subjective impressions on the part of the surgeons, since there is no evidence or published data to indicate that patient compliance with radiation therapy is low.

The findings indicated that gender had no effect on the performance of BCS. This may be due to the small number of female surgeons in this study. Similarly, in a study comparing rates of BCS between male and female surgeons, no statistically significant difference was found between the two groups [11]. However, it is interesting that in our study, 14 out of the 75 male surgeons (19%) indicated that they routinely perform BCS, while 3 out of the 8 female surgeons (38%) stated that they performed BCS.

We found that the surgeons' age and work experience was not significantly associated with performing BCS; other studies have shown that younger surgeons are more likely to perform BCS. In one retrospective study that reviewed the medical records of 952 patients, it was found that surgeons who graduated after 1960 performed BCS more frequently than those who graduated before 1960 [12]. Another study showed that surgeons who graduated after 1980 performed BCS at a significantly higher rate than those who graduated before 1961 [13]. However, the mean age of our study sample was 55.2 (SD = 12.3) years and the mean duration of work experience was 23.2 years (SD = 12.7). In other words, most of our participants were old; only 17% were relatively young. This might explain why most surgeons in this study stated that they performed mastectomy. This is also consistent with the fact that mastectomy is the most prevalent surgical therapy for breast cancer in our teaching hospitals [6,7]. However, the findings from the present study indicate that there is a trend towards more BCS at teaching hospitals, and it is very likely that a bigger sample would show a significant difference between teaching hospitals and private institutes.

There was a statistically significant association between the experience of the surgeons (measured by the number of beast cancer patients treated per year) and performance of BCS. Surgeons treating more than 20 breast cancer patients per year had a statistically significant higher BCS rate (P = 0.007). Few studies have reported a correlation between the surgeons' experience and their breast conservation surgery rates. A population-based study found that patients treated by surgeons with higher breast cancer case-loads were more likely to receive breast conservation therapy [14]. A survey of surgeons carried out in 1986 showed no evidence of a relationship between the number of breast surgery operations performed and the rate of conservative surgery; but in a repeat survey in 1990, when the overall conservative surgery rate had risen to 42%, a significant association was found for surgeons treating 20 or more patients per year [13].

In this study, the most common reasons given for avoiding BCS were uncertainty about the results of breast conservation therapy (46.3%), uncertainty about the quality of available radiotherapy services (32%), and probability of patient non-compliance (32%). Other reasons noted by the surgeons were the higher cost of breast conserving therapy (17.9%), non-availability of radiotherapy facilities (25.4%), and insufficiency of experience with the BCS technique (14.9%). A few studies have reported surgeons' reasons for avoiding BCS. In one study, the majority of surgeons believed that long-term disease-free survival was equal for mastectomy and BCS, and the preference for mastectomy was mostly due to the inconvenience of radiotherapy [15].

## Conclusion

In conclusion, considering the study limitations (e.g. the sample size was small, the surgeons were predominantly old and only eight of them were female), the findings suggest that the overall performance of BCS in Iran is low. In order to increase the rate of conservation therapy there is need to study the outcomes of breast conservation therapy in Iran to determine overall and disease-free survival and local recurrence rates. At the same time the quality of available radiotherapy services should be evaluated. Perhaps this information might help to improve breast cancer outcomes.

## Competing interests

The author(s) declare that they have no competing interests.

## Authors' contributions

MN contributed to the study design, data collection and analysis, and wrote the first draft. ME contributed to the study design and data analysis. AK contributed to the study design and data collection. EH contributed to the data collection. AM contributed to the data analysis and wrote the final draft.

## Pre-publication history

The pre-publication history for this paper can be accessed here:



## Figures and Tables

**Table 1:** The characteristics of the study sample

	**Number**	**%**
**Age (years)**		
<40	14	17
40–59	37	45
≥60	32	38
Mean (SD)	55.2 (12.3)	
**Gender**		
Female	8	10
Male	75	90
**City of practice**		
Tehran (the capital)	20	24
Other cities	63	76
**Work experience (years)**		
≤10	20	24
>10	63	76
Mean (SD)	23.2 (12.7)	
**Occupation status**		
Working at a teaching hospital.	21	25
Working at a public/private hospital	62	75
**Breast cancer case load (patient/per year, n = 70)**		
≤20	56	80
>20	14	20
**Preferred type of surgery for breast cancer**		
Mastectomy	67	81
Breast conserving surgery	16	19

**Table 2:** Reasons stated by the surgeons for avoiding conservative therapy*

	**Number**	**% of the study sample**
Uncertainty about results of conservative therapy	32	46.3
Uncertainty about radiotherapy services	22	32.8
Probability of patient incompliance	22	32.8
Unavailability of radiotherapy services	17	25.4
Higher cost of conservative therapy	12	17.9
Lack of enough experience for performing BCS	10	14.9

* The total exceeds the sample size since each respondent could state several reasons.

**Table 3:** Association between breast cancer practice and the respondents' characteristics

	**Breast conservation No. (%)**	**Mastectomy No. (%)**	**P value**
**Gender**			0.35
Male	14 (82)	61 (92)	
Female	3 (18)	5 (8)	
**Age (years)**			0.60
<40	4 (24)	10 (15)	
40–59	6 (35)	31 (47)	
≥60	7 (41)	25 (38)	
**Work experience**			1.0
≤10	4 (24)	16 (24)	
>10	13 (76)	50 (76)	
**Working at a teaching hospital**			0.12
Yes	7 (41)	14 (21)	
No	10 (59)	52 (79)	
**Breast cancer case load of the surgeon (n = 70)**			0.007
≤20	8 (53)	48 (87)	
>20	7 (47)	7 (13)	
